# Nitrogen and Boron Co-Doped Biochar-Activated Peroxymonosulfate for Degradation of Tetracycline: Performance and Mechanisms

**DOI:** 10.3390/toxics14070627

**Published:** 2026-07-17

**Authors:** Zhitao Tang, Rongkui Su, Chuansheng Chen, Yiting Luo, Mingli Chen, Shunhong Huang, Xiancheng Ma

**Affiliations:** 1 School of Ecology and Environment, Central South University of Forestry and Technology, Changsha 410004, China; 2 College of Mechanical and Intelligent Manufacturing, Central South University of Forestry and Technology, Changsha 410004, China

**Keywords:** sodium lignosulfonate, utilization of waste resources, heteroatom doping, mechanism

## Abstract

Antibiotic residues pose a serious threat to environmental safety. In this study, nitrogen/boron co-doped biochar (NBLB) was successfully prepared using lignosulfonate, an industrial byproduct, as the precursor via a one-step impregnation–pyrolysis method. NBLB was applied to activate peroxymonosulfate (PMS) to degrade tetracycline (TC) in water. The results showed that NBLB-activated PMS degraded 93.4% of TC within 60 min, which was 42.6%, 20.4%, and 27.8% higher than those of undoped biochar, nitrogen-doped biochar, and boron-doped biochar, respectively. Additionally, the NBLB/PMS system exhibited high tolerance to common aqueous anions such as Cl^−^, $CO_{3}^{2-}$, ${\mathrm{PO}}_{4}^{3-}$, ${\mathrm{NO}}_{3}^{-}$ and $SO_{4}^{2-}$. BET tests indicated that the specific surface areas of undoped, N-doped, B-doped, and NBLB were 133.98, 280.22, 304.13, and 342.35 m^2^ g^−1^, respectively, exhibiting a significant enhancement in the specific surface area of NBLB. The co-doping of N and B constructed a hierarchical pore structure, providing an efficient dispersion platform for active sites such as C=O, BC_2_O, and pyridinic-N. Quenching experiments and EPR detection demonstrated that the degradation of tetracycline (TC) in the NBLB/PMS system followed a synergistic oxidation mechanism dominated by ^1^O_2_ and $O_{2}^{•-}$, supplemented by ^•^OH and $SO_{4}^{•-}$. This study prepared high-performance nonmetallic catalysts through a solid waste resource utilization strategy, providing a green and feasible path for the treatment of antibiotic pollution.

## 1. Introduction

Tetracyclines (TCs), as typical broad-spectrum antibiotics, are widely applied in the prevention and treatment of human and animal diseases, as well as livestock breeding [1,2,3]. After entering organisms, TCs are poorly metabolized, and a large number of parent compounds are discharged into the aquatic environment via feces and urine, causing persistent pollution and ecological risks [4,5,6]. As an innovative remediation strategy for water contamination, persulfate-driven advanced oxidation processes (SR-AOPs) have gained increasing attention, outperforming conventional approaches such as adsorption, biological treatment, and membrane filtration. The activation of peroxymonosulfate (PMS) and peroxydisulfate (PDS) enables SR-AOPs to produce both radical and non-radical reactive oxygen species, thereby achieving efficient degradation and mineralization of organic contaminants [7]. SR-AOPs possess the advantages of strong oxidation capacity, low cost, broad environmental adaptability and simple operation [8]. Therefore, SR-AOPs have drawn extensive research attention to the remediation of refractory antibiotic contaminants in aqueous systems, offering novel and viable technical strategies for aquatic ecological restoration [9,10].

In recent years, biomass-derived porous carbon materials have garnered widespread interest by virtue of their low cost, eco-friendly nature, and superior adsorption capacity [11]. Compared with straw-, sawdust- and alkali lignin-derived biochar, sodium lignosulfonate presents superior precursor advantages. Lignosulfonate (LS) features a porous framework, large specific surface area, and a high density of surface oxygen-containing functional groups. Its inherent sulfonate and sodium ions allow in situ self-activation during pyrolysis, constructing hierarchical porous structures without additional activators. Natural S and Na co-doping endows biochar with rich active sites and excellent electron transfer performance [12]. Moreover, as a low-cost and readily available papermaking byproduct, it achieves favorable preparation economy. The porous architecture and surface functional groups of lignosulfonate biochar (LB) are capable of effectively activating PMS and accelerating its decomposition, thus generating reactive oxygen species (ROS) [13], thereby achieving efficient degradation of organic pollutants in aqueous solutions [14]. Despite the fact that LB can activate PMS for the decomposition of organic contaminants, it suffers from shortcomings of limited active sites and weak redox capability. Accordingly, the modification of LB is essential to improve its catalytic performance toward organic pollutant degradation. In this context, recycling waste lignosulfonate into modified biochar for PMS activation offers a feasible and promising route to eliminate organic contaminants in water [15].

Compared with metal-doped carbon, nonmetal-doped biochars lower secondary contamination risks without transition metal ion leaching [16,17]. Therefore, nonmetallic doping has gradually attracted the attention of researchers [18]. Nonmetallic heteroatom doping (B, N, S, P) can effectively regulate the physicochemical properties and surface electronic structure of biochar [19,20]. Due to the similar atomic radius to carbon, B and N atoms can substitute carbon lattices during pyrolysis, forming active configurations (e.g., pyridinic N and graphitic N) and creating abundant edge defects and catalytic active sites [21]. This process induces more defective edges and active sites, and regulates the electronic structure and chemical stability of the carbon surface [22]. For example, B/N co-doped biochar (BC-NB) prepared by two-step pyrolysis could eliminate 92.7% of sulfamethoxazole (SMX) in 30 min [23]. The high-performance B/N co-doped biochar prepared by hydrothermal pyrolysis achieved efficient and rapid degradation of oxytetracycline, and possessed favorable stability in various water matrices, as well as in the presence of typical coexisting ions [24]. Nevertheless, traditional multi-step N/B-doping approaches face cumbersome procedures, irregular heteroatom dispersion and loosely anchored dopants. Differing from sulfur-free precursors, lignosulfonate-derived inherent-S cooperates with N and B to rearrange surface electrons. Herein, lignosulfonate is adopted to realize one-pot in situ B/N co-doping. The synergy between intrinsic-S and external B-N forms well-distributed defective carbon, which endows our biochar with stabilized dopant configuration and enhanced PMS activation compared with multi-step counterparts.

In this work, nitrogen/boron co-doped biochar (NBLB) derived from lignosulfonate was fabricated via a one-step impregnation–pyrolysis route, with industrial waste lignosulfonate employed as the precursor, achieving high-value recycling of industrial solid waste. Synchronous N and B co-doping synergistically modulates the electronic properties of carbon, generates abundant defects and hierarchical pores, and remarkably boosts peroxymonosulfate activation performance. The main research aims are as follows: (1) investigate the reaction rate, adsorption performance and pH adaptability of the NBLB/PMS system toward tetracycline (TC) and clarify the promotion effect and underlying intrinsic mechanism of nitrogen and boron co-doping on the PMS activation performance of pristine lignosulfonate biochar; (2) evaluate the adaptability of the NBLB/PMS system for TC degrading under diverse environmental conditions; and (3) identify the dominant radical and non-radical reactive oxygen species in the reaction system through quenching experiments combined with EPR characterization. This study proposes a feasible strategy to optimize the catalytic activity and stability of biochar catalysts via N and B co-modification, which provides a green and sustainable approach for the elimination of antibiotic contaminants in aqueous environments.

## 2. Materials and Methods

### 2.1. Materials

Sodium lignosulfonate (LS, purity 96%) was purchased from Sinopharm Chemical Reagent Co., Ltd. (Shanghai, China). Tetracycline (TC, AR) and furfuryl alcohol (FFA, AR) were purchased from Aladdin Biochemical Technology Co., Ltd. (Shanghai, China). Urea (CO(NH_2_)_2_, AR), boric acid (H_3_BO_3_, AR), manganese chloride tetrahydrate (MnCl_2_·4H_2_O, AR), peroxymonosulfate (PMS, AR), hydrochloric acid (HCl, AR), methanol (MeOH, AR), tert-butanol (TBA, AR), sodium hydroxide (NaOH, AR), ascorbic acid (AA, AR), sodium carbonate (Na_2_CO_3_, AR), sodium chloride (NaCl, AR), sodium sulfate (Na_2_SO_4_, AR), sodium nitrate (NaNO_3_, AR), and sodium phosphate (Na_3_PO_4_, AR) were purchased from Macklin Biochemical Co., Ltd. (Shanghai, China). Ultrapure water was employed as the solvent throughout the entire experimental process.

### 2.2. Synthesis Method

Sodium lignosulfonate was placed in a tube furnace and heated to 500 °C at a heating rate of 5 °C/min under a continuous nitrogen atmosphere. The temperature was maintained for 120 min, followed by natural cooling to room temperature. The resulting sample was washed repeatedly with 1 mol/L HCl and then rinsed with deionized water to remove residual hydrochloric acid. After being dried at 60 °C for 24 h, the sample was ground into powder and labeled as LB. Similarly, 1 g of sodium lignosulfonate and 0.5 g of urea were dissolved in 20 mL of deionized water, and the mixture was stirred at 80 °C until the water evaporated to dryness. The subsequent preparation conditions were consistent with those of LB, and the obtained sample was named nitrogen-doped lignosulfonate-derived biochar (NLB). Boron-doped lignosulfonate-derived biochar (BLB) was prepared according to the same procedure as NLB, while boric acid was used to replace urea, and the sample was designated as BLB. Moreover, 1.0 g of sodium lignosulfonate, 0.5 g of urea and 0.5 g of boric acid were dissolved in 20 mL of deionized water and stirred at 80 °C until the water evaporated to dryness. All other preparation parameters were consistent with those of NLB. The as-prepared material weighed 0.33 g with a yield of 33%, which was labeled NBLB.

### 2.3. Pollutant Degradation Experiments

TC degradation experiments were performed in a 400 mL beaker in the dark. Briefly, 0.10 g/L catalyst was added into 100 mL of 10 mg/L TC solution, and the mixture was stirred for 60 min to reach adsorption–desorption equilibrium. Afterwards, PMS was introduced to trigger the reaction. At scheduled time intervals of 0, 5, 10, 20, 30, 40 and 60 min, 2.0 mL of the reaction suspension was withdrawn and immediately filtered through a 0.22 μm membrane filter. The concentration of TC was determined by ultraviolet–visible (UV–Vis) spectrophotometry at the maximum absorption wavelength of 355 nm. The effects of different conditions were investigated, including the presence or absence of PMS (catalyst with/without PMS), operating parameters (PMS, NBLB and TC concentration), and water chemistry (solution pH, anions). Triplicate parallel trials were conducted for every batch reaction, including kinetic degradation, quenching verification and cycling performance tests. Mean concentration and efficiency values, together with standard deviations, are illustrated as error bars throughout all graphs. The degradation rate (η) of TC was calculated according to Formula (1).

(1) $$\eta =(1-Ct/C_{0})\;\times \;100\%$$

The degradation kinetics of tetracycline (TC) were fitted using pseudo-zero-order, first-order and second-order kinetic models. The results showed that the first-order kinetic model exhibited the best fit. Therefore, the reaction rate constant *k* was calculated according to Equation (2).

(2) $$-\ln{(C}_{t}/C_{0})=kt$$

where C_0_ represents the initial concentration and *C_t_* is the concentration of TC at any time *t*.

## 3. Results and Discussion

### 3.1. Physicochemical Properties of Catalysts

**Figure 1:**
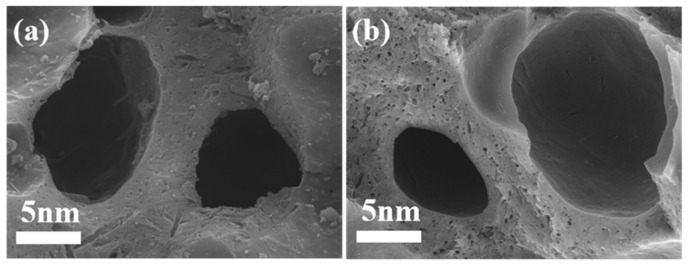
SEM images of LB (**a**) and NBLB (**b**).

The scanning electron microscopy (SEM) images of LB (a) and NBLB (b) prepared with sodium lignosulfonate as the raw material are presented in Figure 1. The pristine LB displays sporadic large pores on its relatively smooth surface, yet sufficient micro- and mesoporous structures are absent. In contrast, NBLB possesses a rougher surface and a richer pore structure. This phenomenon can be attributed to the volatilization of urea during pyrolysis [25], as well as the partial structural collapse of the carbon layers during doping of B and N atoms into the carbon lattice [26], thereby forming a well-developed porous structure.

**Figure 2:**
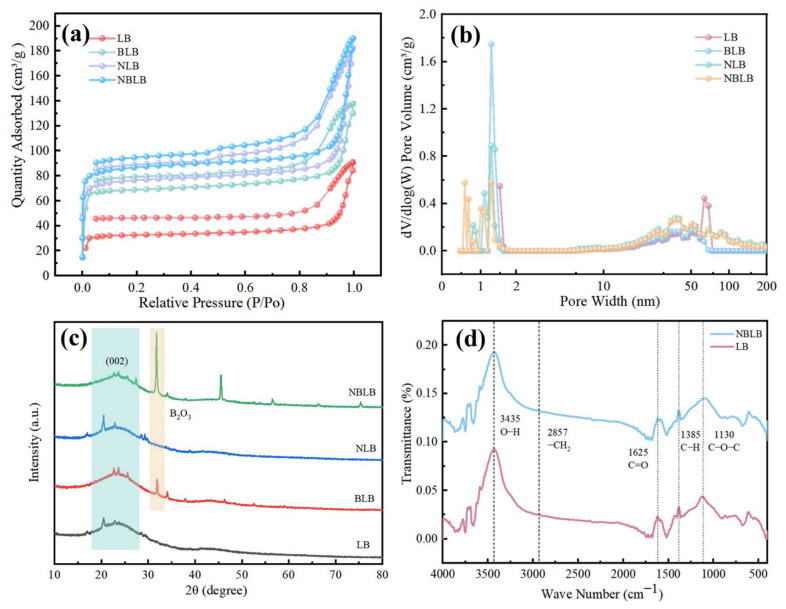
N_2_ adsorption and desorption curves (**a**); pore size distribution (**b**); XRD patterns of LB, BLB, NLB and NBLB (**c**); FTIR spectra of LB and NBLB (**d**).

Brunauer–Emmett–Teller (BET) characterization was further adopted to analyze the pore structure and specific surface area of the prepared catalysts. As displayed in Figure 2a, all samples exhibited typical type IV hysteresis loops in their nitrogen adsorption–desorption isotherms [27], indicating that the as-prepared materials possessed a mesoporous structure. As presented in Table 1, the specific surface areas of LB, BLB, NLB, and NBLB were 133.98, 280.22, 304.13 and 342.35 m^2^ g^−1^. Compared with LB and BLB, the NBLB sample exhibited a significantly larger specific surface area and enhanced adsorption capacity. Figure 2b reveals that N and B co-doping significantly increased the number of micropores in the range of 0.5–1.4 nm of NBLB, improved the mesopore volume in the 20–50 nm range, and optimized the macroporous framework to form a hierarchical micro-, meso- and macroporous architecture. This observation was further supported by the SEM results, demonstrating the more complex porous structure of NBLB.

X-ray diffraction (XRD) patterns of LB, BLB, NLB and NBLB are shown in Figure 2c. All samples exhibited obvious diffraction peaks at 21–25° and 44°, which are assigned to the (002) and (100) crystal planes of graphitic carbon [28]. This result confirmed the typical graphitic structure of the synthesized biochar and the successful formation of graphitic biochar. No obvious shift in diffraction peaks was observed before and after doping, suggesting that N and B doping did not change the intrinsic crystal structure of biochar. In addition, new characteristic peaks at 2θ = 31.7° appeared in BLB and NBLB, which may be associated with the formation of B_2_O_3_, In combination with XPS characterizations, it can be confirmed that boron is successfully incorporated into the biochar framework via pyrolysis treatment [29]. B_2_O_3_ vapor acts as the active agent for the doping reaction, accelerating the formation of oxidized boron species (BCO_2_ and BC_2_O) within the carbon matrix [30]. All oxidized boron species can participate in the activation process of PMS [31]. Doping biochar with N and B heteroatoms can elevate its graphitization degree, which is ascribed to the formation of graphitic N and BC_2_O moieties during doping [32,33]. Fourier transform infrared spectroscopy (FTIR) was applied to investigate the surface functional groups present in LB and NBLB (Figure 2d). The characteristic peak positions of LB and NBLB were basically consistent, indicating that N/B co-doping did not destroy the basic carbon skeleton structure of biochar. The characteristic peaks at 3435 cm^−1^, 2857 cm^−1^, 1625 cm^−1^, 1385 cm^−1^ and 1130 cm^−1^ corresponded to the stretching vibrations of O-H, -CH_2_, C=O, C-H and C-O-C groups [14,34], respectively. These results confirm that abundant oxygen-containing functional groups and sp^2^-conjugated carbon structures exist on the biochar surface. Co-doping of B and N can induce a synergistic interaction between heteroatoms and form a B–N-C composite structure. Although LB also possesses abundant functional groups, its relatively simple pore structure restricts the degradation performance by limiting the number of available active sites [35].

### 3.2. TC Degradation Performance Evaluation

**Figure 3:**
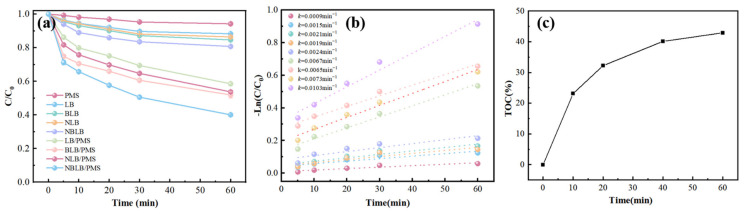
The removal rates of TC of PMS, LB, BLB, NLB, NBLB, LB/PMS, BLB/PMS, NLB/PMS, and NBLB/PMS) (**a**); the pseudo-first-order kinetic model was used for fitting to calculate the reaction rate constants of different systems (**b**); TOC removal efficiency of the NBLB/PMS system (**c**).

Figure 3a shows the TC removal efficiencies by single and coupled systems of LB, BLB, NLB, NBLB, LB/PMS, BLB/PMS, NLB/PMS and NBLB/PMS. Without pH adjustment, the TC removal efficiencies of the single PMS, LB, BLB, NLB and NBLB systems within 60 min were 5.75%, 11.70%, 15.35%, 13.55% and 19.25%, respectively. PMS alone exhibited almost no removal efficiency of TC. N and B modification improved the adsorption capacity of biochar, while the adsorption performance of the four materials toward TC was relatively limited. For the coupled systems of LB/PMS, BLB/PMS, NLB/PMS and NBLB/PMS, the TC removal efficiencies reached 41.45%, 48.10%, 46.25% and 59.90%, respectively. Compared with the LB/PMS, BLB/PMS, and NLB/PMS systems, the NBLB/PMS system increased the TC removal efficiency by 42.58%, 22.87% and 27.78%, respectively. The removal efficiency of each binary system was notably higher compared to that of the single systems, which demonstrates that NBLB is capable of efficiently activating PMS to produce abundant reactive species for the oxidative degradation of TC [36,37]. Moreover, TOC mineralization performance of the system was evaluated via post-reaction solution TOC detection (Figure 3c). The mineralization efficiency surged quickly before leveling off, achieving a 43% TOC removal rate. This confirms that the system can effectively mineralize most organic matter into carbon dioxide, water and other inorganic salts.

### 3.3. Impacts of Environmental Factors on TC Degradation in the NBLB/PMS System

**Figure 4:**
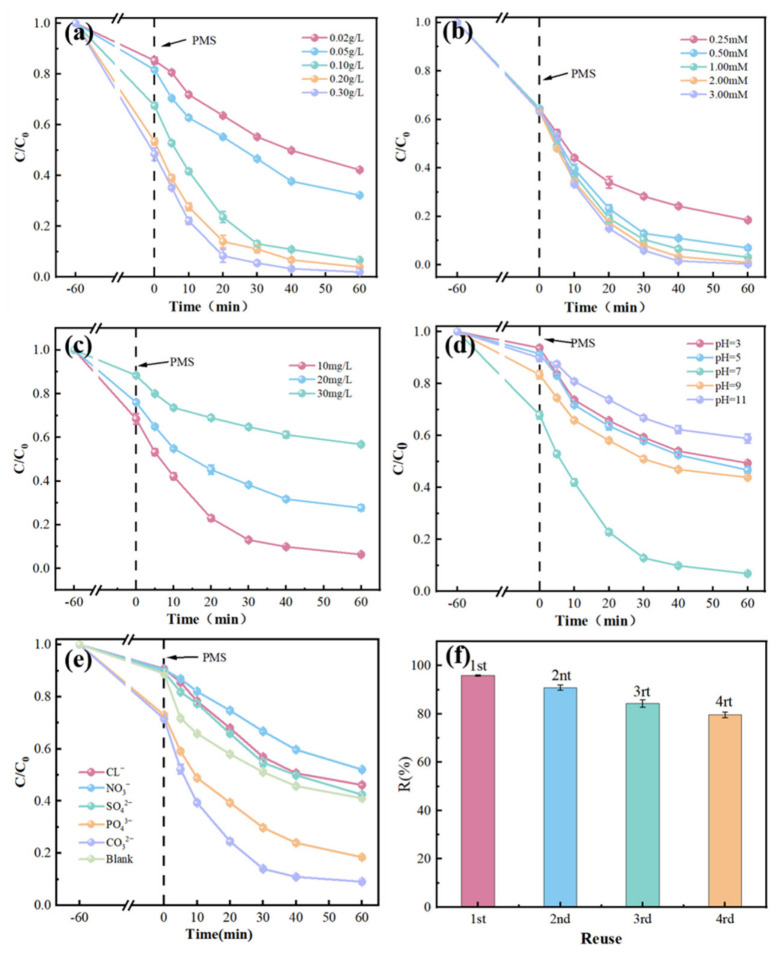
NBLB dose (**a**); influence of PMS concentration (**b**), influence of TC concentration (**c**), and influence of solution pH (**d**) on the degradation rates of TC in the NBLB/PMS system; the zeta potential of NBLB at different pH; the removal rates of influence of anionic species (**e**); the cycling experiments in the NBLB/PMS system for four cycles (**f**).

The influences of catalyst dosage, PMS dosage, initial TC concentration, solution pH and coexisting anions on TC degradation within the NBLB/PMS system were systematically explored, and these findings are summarized in Figure 4. The influences of NBLB dosage (0.02–0.30 g/L) and PMS concentration (0.25–3.00 mM) on TC degradation are presented in Figure 4a,b, respectively. The degradation efficiency of TC increased with the increase in PMS concentration and NBLB dosage. Specifically, with the PMS concentration increasing from 0.25 mM to 1.00 mM, the TC removal efficiency increased correspondingly from 81.55% to 96.90%. Further elevating the PMS concentration to 2.00 mM and 3.00 mM showed negligible promotion of removal efficiency. PMS concentration above 1.00 mM exerted no prominent promotion of TC degradation, mainly due to lowered oxidant utilization [38] and the self-quenching effect of reactive radicals [39]. As the NBLB dosage increased from 0.02 g/L to 0.30 g/L, the TC degradation efficiencies within 60 min were 57.78%, 67.75%, 93.34%, 96.05% and 98.05%, respectively. The enhanced removal efficiency at higher catalyst dosage was attributed to the availability of more active sites [40,41]. Overall, moderately increased PMS concentration and catalyst dosage facilitated the removal of target pollutants in the catalytic system. A higher PMS concentration could generate more reactive species and thus accelerate TC degradation [39,42]. Nevertheless, excessive catalyst dosage would decrease material utilization and raise operational costs [39]. To achieve efficient TC removal with acceptable economic cost, the optimal operating parameters were determined as a PMS concentration of 1.00 mM and NBLB dosage of 0.10 g/L.

Figure 4c presents the influence of initial TC concentration. As the initial concentration of TC increased in the range of 10 mg/L to 30 mg/L, its degradation efficiency declined from 93.66% to 43.25%. This phenomenon was mainly ascribed to the insufficient availability of reactive species for pollutant oxidation with the increase in contaminant loading [35]. Solution pH is a critical parameter regulating PMS activation. The TC degradation efficiency of the NBLB/PMS system at various initial pH values within 60 min is depicted in Figure 4d. Solution pH was tuned to 3, 5, 7, 9 and 11 by using 0.1 mol/L HCl and NaOH. The corresponding TC degradation efficiencies were 50.61%, 53.28%, 93.14%, 56.13% and 41.36%, respectively. TC degradation was suppressed at pH 3 and 5, which can be explained by the establishment of intense hydrogen bonds between H^+^ and PMS peroxy bonds [43]. Under alkaline conditions (pH = 9~11), PMS could react with ${\mathrm{OH}}^{-}$ to generate low-activity species [44]. In general, the NBLB/PMS system achieved the optimal TC removal efficiency at neutral pH = 7.

Widely distributed anions in natural water bodies, including Cl^−^, ${\mathrm{CO}}_{3}^{2-}$, ${\mathrm{PO}}_{4}^{3-}$, ${\mathrm{NO}}_{3}^{-}$ and ${\mathrm{SO}}_{4}^{2-}$, could exert an influence on TC degradation in the NBLB/PMS system. In this study, 10 mM of each anion was separately added into the reaction system for comparison with the blank group. Figure 4e shows a weak inhibitory influence of Cl^−^ on TC degradation, which originates from the reaction between chloride ions and reactive radicals to yield Cl· and ${\mathrm{Cl}}_{2}^{\cdot -}$ [45,46], thereby achieving partial degradation of TC. ${\mathrm{NO}}_{3}^{\cdot -}$ showed a slight inhibitory effect on TC degradation, which was attributed to its reaction with ^•^OH and other reactive radicals generated in the AOP system to form low-activity species [47,48]. ${\mathrm{SO}}_{4}^{2-}$ had almost no obvious impact on the degradation process. Nevertheless, the addition of ${\mathrm{CO}}_{3}^{2-}$ significantly promoted TC degradation, with the degradation efficiency increasing from 58.7% (Blank) to 81.8%. Similarly, ${\mathrm{PO}}_{4}^{3-}$ also remarkably facilitated TC degradation with an efficiency of 89.7%. The promotion mechanism was mainly that ${\mathrm{CO}}_{3}^{2-}$ and ${\mathrm{PO}}_{4}^{3-}$ could regulate the solution’s pH through hydrolysis, thereby enhancing the TC degradation efficiency.

To assess the reusability and catalytic stability of NBLB for PMS activation, four consecutive cyclic tests were carried out. After the first TC degradation process, the catalyst underwent separation, recovery, filtration and drying. The regenerated sample was then placed into TC solution to conduct the second batch of degradation reactions under consistent conditions: [TC] = 10 mg/L, [catalyst] = 0.10 g/L, and [PMS] = 1 mM. As illustrated in Figure 4f, the TC removal efficiency was maintained at 79.6% after four consecutive cycles. These results confirm that NBLB can act as a sustainable catalyst for PMS activation and antibiotic degradation in aqueous environments.

### 3.4. TC Removal Mechanism

**Figure 5:**
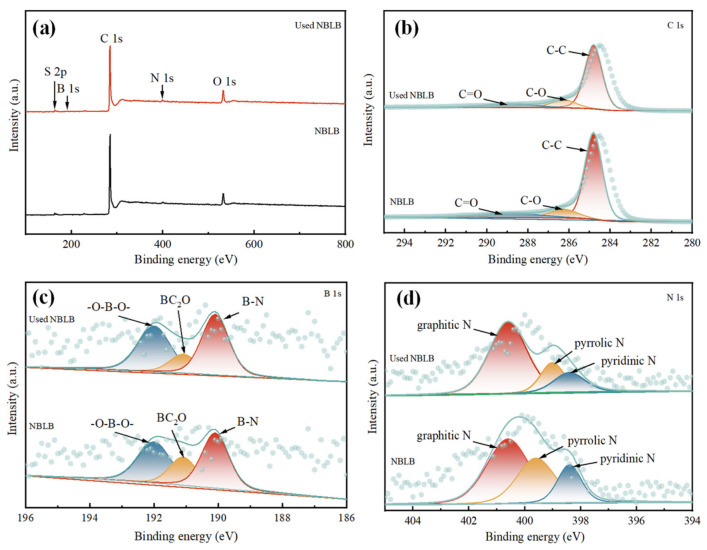
XPS survey spectra (**a**) of NBLB and used NBLB, and C 1s (**b**), B 1s (**c**), and N 1s (**d**) spectra of NBLB and used NBLB.

In order to explore the surface-active sites of NBLB toward PMS activation, the X-ray Photoelectron Spectroscopy (XPS) spectra of NBLB before and after reaction were comparatively investigated. It can be seen from Figure 5a that five elements, including C, O, B, N and S, are detected in the sample [14], indicating the successful incorporation of N and B onto the NBLB surface. The full XPS survey spectrum of NBLB exhibited characteristic peaks at 284.8 eV for C 1s and 531.0 eV for O 1s, alongside obvious signals of N 1s (400.1 eV) and B 1s (191.1 eV) [34]. The C 1s spectrum of NBLB can be deconvoluted into three component peaks, corresponding to C-C, C-O, and C=O [49,50] (Figure 5b). The content of C=O decreased from 12.78% to 12.47% after the reaction. As a catalytic site for PMS activation, C=O generates ROS through two pathways: (1) The electrophilic C=O on NBLB, which possesses excellent electron transfer capability. It can interact with PMS and capture electrons to generate singlet oxygen (^1^O_2_). (2) The lone electron pairs of C=O groups on NBLB serve as Lewis base sites, which elevate the electron density of neighboring B and N atoms and further accelerate the redox reaction process [51,52].

The XPS spectrum of B 1s for NBLB can be deconvoluted into three components: B-N (190.1 eV, 41.58%), BC_2_O (191.1 eV, 21.22%), and -O-B-O- (192.0 eV, 37.20%) [34] (Figure 5c). These species are formed by the substitution of carbon atoms at trigonal lattice sites by boron atoms. The BC_2_O groups on NBLB can modulate and enhance electronic properties. Following the reaction, there was a marked decrease in the relative content of BC_2_O, which fell to 11.41%. These results imply a possible correlation between BC_2_O configurations and the modulation of carbon charge distribution and redox properties during PMS activation. This observation is due to the adsorption of PMS onto BC_2_O active sites, effectively improving the catalytic activity of the NBLB/PMS system [53]. The deconvolution of the N 1s XPS spectrum of NBLB yields three peaks, assigned to graphitic N (400.6 eV, 51.3%), pyrrolic N (399.6 eV, 31.83%), and pyridinic N (398.4 eV, 16.87%) (Figure 5d). This indicates that nitrogen atoms are incorporated into the graphitic layered framework of biochar via carbon substitution, and this doping mode effectively improves the electronic conductivity of biochar [45]. Prior to and following the reaction, the relative content of graphitic N increased from 51.3% to 67.41%, while pyrrolic N decreased from 31.83% to 17.60% and pyridinic N declined from 16.87% to 14.99%. This implies that pyrrolic N and pyridinic N may act as potential active moieties that partially accelerate electron transfer during PMS activation [54]. These variations reveal a profound surface structural reconstruction of the catalyst during PMS activation. The elevated proportion of graphitic N not only strengthens the electron conduction capability of the material [55] but also stabilizes the B-N-C active configuration, endowing the catalyst with excellent reusability and intrinsic catalytic activity.

In summary, BC_2_O and pyrrolic N serve as the predominant active sites responsible for PMS activation. The coexistence of BC_2_O and pyridinic N can further elevate the overall charge density of carbon-based catalysts, optimize the adsorption and activation efficiency of PMS, and enhance the electron-donating capability of the catalysts. The activated conjugated π electrons can migrate to the adsorbed peroxymonosulfate, thereby facilitating the cleavage of the peroxy bond in peroxymonosulfate and promoting the formation of free radicals [30]. The BC_2_O on NBLB could function as a Lewis acidic group to facilitate the adsorption and activation of PMS and further generate ^1^O_2_, with NBLB serving as an electron mediator [56].

### 3.5. Detection and Identification of ROS

**Figure 6:**
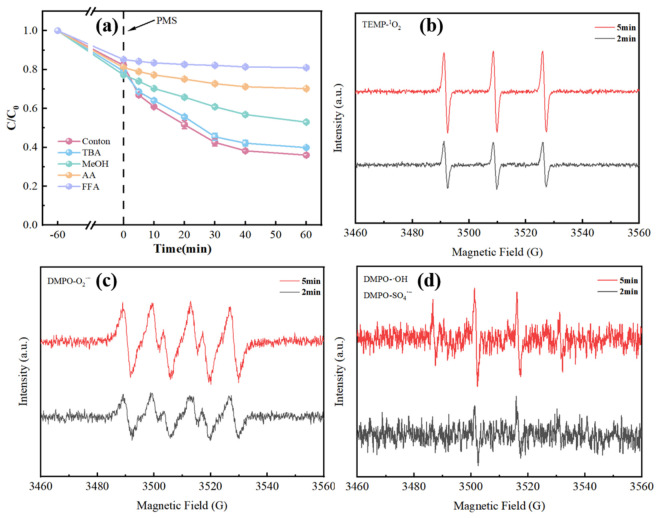
(**a**) Effect of FFA, AA, TBA and MeOH on TC degradation in the NBLB/PMS system. (**b**) EPR signals of PMS and NBLB/PMS from TMPO-^1^O_2_, (**c**) DMPO-$O_{2}^{•-}$, (**d**) DMPO-^•^OH and DMPO-$SO_{4}^{•-}$.

To elucidate the contribution of reactive oxygen species to TC degradation in the NBLB/PMS system, tert-butanol (TBA) and furfuryl alcohol (FFA) were employed as specific scavengers for ^•^OH and ^1^O_2_, respectively. Methanol (MeOH) was applied to quench ^•^OH and $SO_{4}^{•-}$, while ascorbic acid (AA) was used for $O_{2}^{•-}$ radical elimination. As shown in Figure 6a, AA (0.5 mM), FFA (0.5 mM), TBA (10 mM) and MeOH (10 mM) were separately added into the NBLB/PMS system. Neither MeOH nor TBA showed a remarkable inhibitory effect on TC degradation, whereas AA and FFA exhibited a remarkable inhibitory effect. This result indicates that ^1^O_2_ and $O_{2}^{•-}$ served as the predominant active species in the NBLB/PMS reaction system, followed by ^•^OH and $SO_{4}^{•-}$. To directly verify the formation of these reactive species, EPR characterization was performed using TEMP and DMPO as spin-trapping agents for ^1^O_2_, $O_{2}^{•-}$, $SO_{4}^{•-}$ and ^•^OH, which can form the adducts of TEMP-^1^O_2_, DMPO-$O_{2}^{•-}$, DMPO-$SO_{4}^{•-}$ and DMPO-^•^OH, respectively. As displayed in Figure 6b, a distinct 1:1:1 triplet signal was detected when TEMP was adopted as the trapping agent for ^1^O_2_, confirming the abundant production of ^1^O_2_ in the reaction system. In Figure 6c, typical DMPO-$O_{2}^{•-}$ radical adduct signals were successfully captured in the NBLB/PMS system, directly confirming the formation of $O_{2}^{•-}$. As shown in Figure 6d, the characteristic quartet peaks of DMPO-^•^OH and weak DMPO-$SO_{4}^{•-}$ radical signals were simultaneously observed. The direct EPR results are in good agreement with the indirect inference from quenching experiments. The degradation of tetracycline (TC) proceeds through a complex oxidation mechanism, in which ^1^O_2_ and $O_{2}^{•-}$ act as the primary contributing reactive species, with the synergistic participation of $SO_{4}^{•-}$ and ^•^OH.

### 3.6. Preliminary Assessment of Microbial Toxicity

**Figure 7:**
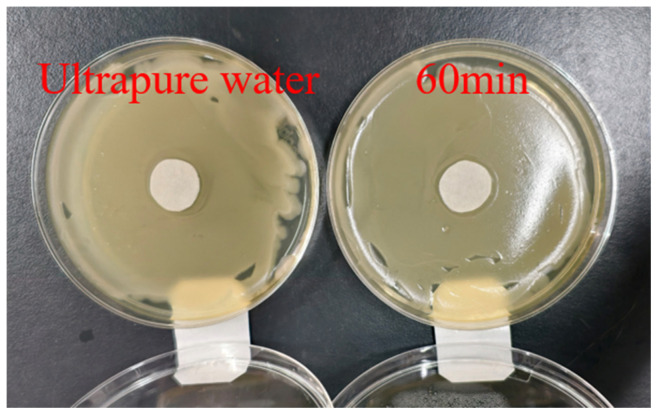
Antibacterial toxicity of ultrapure water after 60 min reaction with the NBLB/PMS system against *Escherichia coli*.

This study used standard slant strains of Escherichia coli as the test organism. With pre-prepared nutrient agar plates and LB medium, the paper disk diffusion method was applied to perform inhibition zone assays, so as to qualitatively analyze the antibacterial toxicity of 10 mg/L tetracycline against *E. coli* (Figure 7) [57,58]. The results showed that the inhibition zones of tetracycline after 60 min of degradation were almost indistinguishable from those of ultrapure water, which confirmed that the antibacterial toxicity of tetracycline at this concentration was nearly eliminated after degradation. This experiment achieved a qualitative evaluation of tetracycline’s antibacterial effect and can serve as a reference for studying the fundamental antibacterial characteristics of antibiotics and conducting preliminary assessments of the microbial toxicity of tetracycline in the environment.

## 4. Conclusions

NBLB derived from lignosulfonate was successfully synthesized through a one-step impregnation–pyrolysis strategy. Heteroatom modification effectively optimized the pore structure and enlarged the specific surface area of the pristine biochar, which further endowed the material with abundant surface-active sites. NBLB exhibited excellent catalytic activity for activating PMS. Under optimal reaction conditions, the degradation efficiency of TC achieved 93.4% within 60 min. After four successive recycling tests, the TC removal rate remained at 79.6%, verifying the favorable reusability and stable catalytic performance of NBLB in actual complex water environments. The active sites responsible for PMS activation on NBLB mainly included C=O groups, BC_2_O groups, pyridinic N, and defect structures. The N and B co-doping strategy constructed a hierarchical pore structure (micro-meso-macropores) in NBLB. The large specific surface area of NBLB not only provided an efficient dispersion platform for C=O, BC_2_O, and pyridinic N active sites, but also ensured the rapid diffusion of PMS and TC molecules, while offering strong adsorption sites for target pollutants. Quenching experiments and EPR characterization confirmed that the TC degradation in the NBLB/PMS system followed a synergistic oxidation mechanism dominated by singlet oxygen ^1^O_2_ and $O_{2}^{•-}$, with the auxiliary participation of ^•^OH and $SO_{4}^{•-}$. Using industrial waste lignosulfonate as the raw material to prepare NBLB catalyst achieves low cost and no metal leaching, which delivers both environmental and practical application merits. This research proposes a novel catalytic material derived from solid waste, which can be applied for the treatment of antibiotic wastewater through advanced oxidation processes.

## Figures and Tables

**Table 1:** The pore volume and specific surface area of the target samples.

Materials	BET Surface Area (m^2^ g^−1^)	Average Pore Size (nm)	Pore Volume (cm^3^ g^−1^)	
			V_total_	V_mic_
LB	133.98	30.97	0.1315	0.0220
BLB	280.22	19.74	0.1679	0.0050
NLB	304.13	25.94	0.2599	0.0218
NBLB	342.35	21.81	0.2504	0.0193

V_total_ and V_mic_ represent the total volume and the volume of micropores, respectively.

## Data Availability

The raw data supporting the conclusions of this article will be made available by the authors upon request.

## Abbreviations

The following abbreviations are used in this manuscript:

TC	Tetracycline
PMS	peroxymonosulfate
LB	Sodium lignosulfonate derived biochar
NBLB	N, B co-doped sodium lignosulfonate biochar
SEM	Scanning electron microscope
XRD	X-ray diffraction
FTIR	Fourier transform infrared spectroscopy
BET	Brunauer–Emmett–Teller
XPS	X-ray photoelectron spectroscopy
EPR	Electron paramagnetic resonance
